# The Association of Specific Dietary Patterns with Cardiometabolic Outcomes in Women with a History of Gestational Diabetes Mellitus: A Scoping Review

**DOI:** 10.3390/nu15071613

**Published:** 2023-03-27

**Authors:** Hannah O’Hara, Josh Taylor, Jayne V. Woodside

**Affiliations:** 1 Centre for Public Health, School of Medicine, Dentistry and Biomedical Sciences, Queen’s University Belfast, Belfast BT12 6BJ, UK; 2 Belfast Health and Social Care Trust, Belfast BT12 6BJ, UK; 3 Institute for Global Food Security, Queen’s University Belfast, Belfast BT12 6BJ, UK

**Keywords:** gestational diabetes mellitus, dietary pattern, type 2 diabetes, cardiovascular risk

## Abstract

Gestational diabetes mellitus is associated with a significantly increased risk of later type 2 diabetes (T2DM) and cardiovascular disease (CVD). Post-natal interventions aim to reduce this risk by addressing diet and lifestyle factors and frequently focus on restricting energy or macronutrient intake. With increased interest in the role of complete dietary patterns in the prevention of cardiometabolic disease, we sought to evaluate what is known about the role of dietary patterns in reducing cardiometabolic risk in women with previous GDM. A systematic search was conducted to identify studies relating to dietary pattern and cardiometabolic parameters in women with a history of GDM. The search criteria returned 6014 individual studies. In total, 71 full texts were reviewed, with 24 studies included in the final review. Eleven individual dietary patterns were identified, with the Alternative Health Eating Index (AHEI), Mediterranean diet (MD), and low glycaemic index (GI) as the most commonly featured dietary patterns. Relevant reported outcomes included incident T2DM and glucose tolerance parameters, as well as several cardiovascular risk factors. Dietary patterns which have previously been extensively demonstrated to reduce the risk of cardiovascular and metabolic disorders in the general population, including AHEI, MD, and DASH, were found to be associated with a reduction in the incidence of T2DM, hypertension, and additional risk factors for cardiometabolic disease in women with a history of GDM. Notable gaps in the literature were identified, including the relationship between dietary patterns and incident CVD, as well as the relationship between a low GI diet and the development of T2DM in this population.

## 1. Introduction

Gestational diabetes mellitus (GDM) is a glucose intolerance with the first onset or diagnosis during pregnancy [1]. Well-established risk factors for the condition include overweight and obesity, advanced maternal age, a family history of diabetes, and polycystic ovarian syndrome. The global prevalence of the condition is rising, with around 13% of pregnancies being affected [2]. Gestational diabetes is not only associated with adverse perinatal outcomes but also has significant implications for the future health of the mother. Recent meta-analyses demonstrated that women with a history of GDM are 10 times more likely to develop type 2 diabetes mellitus and twice as likely to develop cardiovascular disease (CVD) later in life [3,4]. Women with previous GDM are also more likely to develop metabolic syndrome later in life [5]. This metabolic syndrome is known to be associated with an increased risk of CVD but there is suggestion that it may be a stronger risk factor in women than in men [6]. Women with a history of GDM are more likely to have hypertension and dyslipidaemia, be taking lipid-modifying medication, and are more likely to have had these conditions diagnosed at an early age [7,8,9]. The increased risk of T2DM and CVD for women with a history of GDM is most pronounced in the first decade post-partum and so interventions aiming to improve modifiable risks factors require early adoption, especially given that CVD has a long subclinical latent period. The post-partum period is, therefore, an important opportunity to introduce interventions to reduce the risk of T2DM and CVD.

One of the strongest risk factors for developing GDM is obesity. Women are at increased risk of weight gain during their reproductive years, and excessive weight gain during pregnancy and a high pre-pregnancy BMI are associated with post-partum weight retention [10,11,12,13,14]. In the post-partum period, a BMI of over 25 kg/m^2^ is associated with a 2–3-fold increased risk of developing T2DM in those with a history of GDM [15]. Failure to lose gestational weight gain by 6 months post-partum is considered an important predictor of obesity and obesity-related conditions, including CVD in subsequent decades [16,17]. Furthermore, post-partum weight gain appears to convey an additional risk of T2DM with higher levels of weight gain associated with a higher risk of T2DM [18,19].

The post-partum period represents a unique opportunity to introduce interventions to reduce the long-term risk of cardiometabolic disease. Commonly employed strategies include those encouraging weight loss and maintenance, increased physical activity, and improved diet quality [19,20,21,22]. Lifestyle interventions that promote increased levels of physical activity in conjunction with healthy eating have been shown to reduce the incidence of T2DM in women with a history of GDM [23,24]. Weight loss in the post-partum period has also been shown to be associated with a reduced risk of T2DM [25]. Weight loss in this group of women has also been demonstrated to improve lipid profiles and fasting glucose [26,27]. Furthermore, there is suggestion that the increased risk of CVD in women with a history of GDM is mediated at least in part by weight gain and a lack of a healthy lifestyle [28,29]. 

Whilst dietary interventions have been shown to reduce obesity and the risk of type 2 diabetes, specific information regarding dietary composition is often lacking. Interventions frequently promote a low-fat or low-calorie approach to dietary modification. Diets which restrict macronutrients/calories, whilst once considered prudent dietary advice, are now considered to be less effective at achieving sustained weight loss and glycaemic control as they are often associated with high levels of refined carbohydrate consumption [30]. There has recently been increased interest in the role of complete dietary patterns in the prevention of T2DM and CVD in order to account for diet heterogeneity and food-nutrient interactions [31,32,33].

### 1.1. Dietary Patterns

Several specific dietary patterns have been described in the literature which have been demonstrated to be associated with a reduced risk of cardiometabolic disorders in the general population, including the Mediterranean diet (MD), Dietary Approaches to Stop Hypertension (DASH) and the Alternative Healthy Eating Index (AHEI) [34,35,36,37,38,39,40,41,42,43,44]. Diets which have a low Glycaemic Index (GI) have also been demonstrated to be beneficial in reducing the incidence of T2DM in the general population and improving blood glucose control [45,46,47]. There is also recent interest in the role of plant-based diets, with evidence suggesting that they are associated with a reduction in the risk of T2DM and insulin resistance [48,49,50].

### 1.2. Dietary Patterns and Prevention of Gestational Diabetes

Several dietary patterns have also been demonstrated to be effective at the prevention of metabolic pregnancy complications and improved blood glucose control in GDM. For example, following a MD before or during pregnancy reduces the risk of developing GDM [51,52]. Higher adherence to the AHEI has also been demonstrated to be associated with a lower risk of GDM [43]. It is clear that there is a role for specific dietary patterns in the prevention of obesity, T2DM, and CVD and that dietary patterns can influence the development of GDM. However, evidence regarding the association between dietary patterns in the post-natal period in women with a history of GDM and the subsequent development of cardiometabolic disorders is less abundant.

Previously conducted reviews have explored the role of dietary interventions in reducing the incidence of T2DM and CVD in women with a history of GDM; however, as previously discussed, details regarding specific dietary advice are often lacking or focus on the restriction of energy intake or specific macronutrients. The objective of this scoping review is to determine whether specific dietary patterns are associated with a reduction in risk of T2DM and CVD in women with a history of GDM and to summarise the literature surrounding the association of dietary patterns with the development of cardiometabolic abnormalities in this cohort. The primary outcomes of interest are T2DM and CVD. Additional outcomes include cardiometabolic risk factors and additional measures of cardiometabolic health. A preliminary search of MEDLINE, the Cochrane Database of Systemic Reviews, and JBI Evidence Synthesis was conducted, and no current or underway systematic reviews or scoping reviews were identified on this topic.

## 2. Materials and Methods

The proposed scoping review was conducted in accordance with the JBI methodology for scoping reviews [53] and considered the evidence relating to women with a history of GDM. Animal studies were excluded and there was no restriction on the age of participants so that the full extent of the evidence would be described. All types of quantitative study designs including experimental, quasi-experimental, and analytical observational studies were considered. For experimental studies of a defined intervention, studies were only included if participant adherence to a specified dietary pattern was determined. The outcomes of interest included T2DM, CVD, and modifiable risk factors for cardiometabolic disease such as weight gain and hypertension. Dietary patterns vary widely according to geographical location and so there were no restrictions on article inclusion based on the geographical area. There was also no restriction on the date of publication.

### 2.1. Search Strategy

The search strategy aimed to locate published and unpublished studies. An initial limited search of MEDLINE was undertaken to identify articles on the topic. The text words contained within the titles and abstracts of relevant articles and the index terms used to describe the articles were used, with the assistance of a subject librarian, to develop a full search strategy for MEDLINE, EMBASE, and Web of Science, adapted for each data source, which was conducted in March 2022. The search terms are given in Appendix A. This meets the criteria for a draft search strategy for at least one database, required in the PRISMA-P checklist and by JBI [53,54]. The reference list for all included articles were also hand-searched for additional relevant articles, completing the three-step search strategy recommended by JBI [53]. The search for unpublished studies included OpenGrey, EThOS, DART-Europe, OATD, and the International Clinical Trial Registry Platform.

Following the search, all identified citations were collated and uploaded into EndNote X8 (Clarivate Analytics, PA, USA) and duplicates were removed. The titles and abstracts were then screened by two independent reviewers (H.OH., JT.) for assessment against the inclusion criteria for the review. Potentially relevant articles were then retrieved in full, and the full text was assessed in detail against the inclusion criteria by two independent reviewers (H.O.H., J.T.). Any disagreements arising between the reviewers were resolved through discussion and with the input of a third reviewer (J.V.W.). An additional search of PubMed was carried out in November 2022 using the search term ‘(gestational diabetes) AND (diet)’ to capture relevant studies which had been published since the initial search. 

### 2.2. Data Extraction

Data were extracted from the included articles by two independent reviewers (H.OH., JT.). The extracted information included study design, date, location, duration, and aims as well as the participant numbers, inclusion and exclusion criteria, and primary and secondary study outcomes. A sample of the extraction form is provided (Appendix B). As above, any disagreements arising between the reviewers were resolved through discussion or involvement of a third reviewer (J.V.W).

## 3. Results

### 3.1. Study Selection

In total, 3521 records were identified from Embase, 877 from Medline, and 3229 from the Web of Science. After removal of the duplicates, 6014 articles were screened. Following the screening, 72 full text articles were reviewed for eligibility for inclusion. One study was reported over three separate articles which were identified by the search. Whilst each article was not individually eligible for inclusion, they were all included as the collated information provided in all of the articles facilitated eligibility [55,56,57]. One article was found to be a secondary analysis of a randomised controlled trial (RCT). The article reporting the original RCT was retrieved in order to assess the eligibility of the study. Both articles were included as the collated information confirmed the eligibility for inclusion [58,59].

From the additional limited search of PubMed, one further relevant article was retrieved which was a cohort study describing the associations between the incremental addition of modifiable risk factors and the risk of T2DM in women with previous GDM [60]. The findings regarding dietary patterns had been reported in an article already included within the review [61] and this additional article was therefore not included.

**Figure 1:**
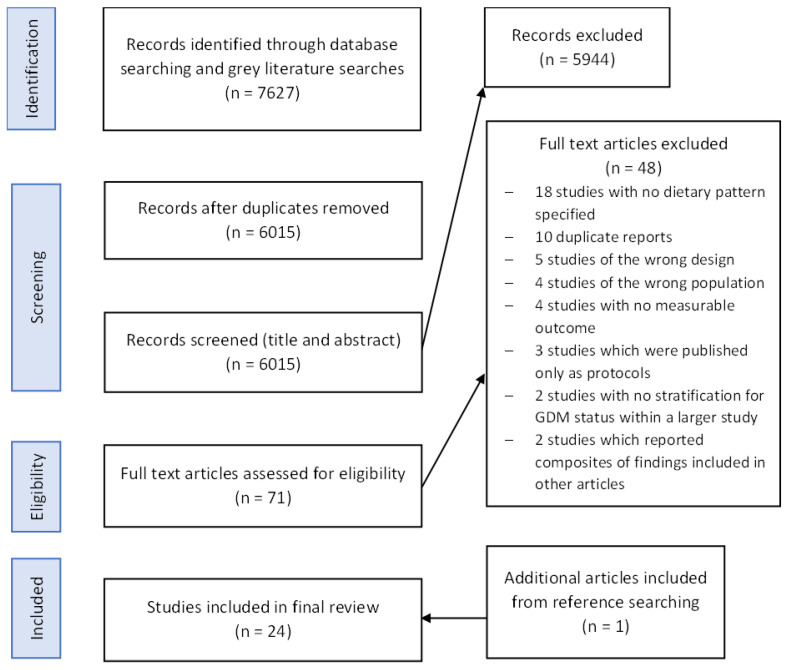
PRISMA flow diagram of identification, screening, eligibility, and inclusion of articles within the scoping review.

Twenty-four articles were included in the final review (Figure 1). The main reasons for study exclusion were the lack of measurement of adherence to the dietary pattern of interest, duplication of findings, and no stated measurable outcome. All studies were published within the last 11 years.

### 3.2. Overview of Studies

The characteristics of the included studies are summarised in Supplementary Table S1. Nine studies were conducted within North America, with seven carried out in the USA and two in Canada. Nine studies were also carried out in Australasia (three in Australia, three in China, two in Malaysia, and one in Korea.) The remainder were carried out in Europe, with one study carried out in each of Germany, Spain, and Italy.

Eleven individual dietary patterns were identified: AHEI, low GI, MD/aMED, DASH, Chinese Healthy Eating Index (CHEI), Australian Recommended Food Score (ARFS), Commonwealth Scientific and Industrial Research Organisation Healthy Diet Score (CSIRO-HDS), Dietary Inflammatory Index (DII), Minimum dietary Diversity for Women (MDD-W), increased plant-based dietary components, and increased fruit and vegetable intake. The most commonly reported dietary patterns were the AHEI (six studies), the MD (five studies), and low GI (five studies). The reported relevant outcomes were highly varied. Five studies set out to determine whether the dietary pattern of interest had any effect on incident T2DM [61,62,63,64,65]. No studies were identified which evaluated the effect of dietary patterns on the incidence of cardiovascular events; however, one study reported carotid intima media thickness as a measure of subclinical atherosclerosis [66]. The remaining studies reported biochemical markers of glucose tolerance and indices of insulin resistance, as well as biochemical indicators of cardiovascular risk including lipid profile, for example. Additional reported outcomes included hypertension and anthropometric indictors of cardiometabolic risk such as BMI and waist circumference.

### 3.3. Overview of Dietary Patterns and Outcomes

**Figure 2:**
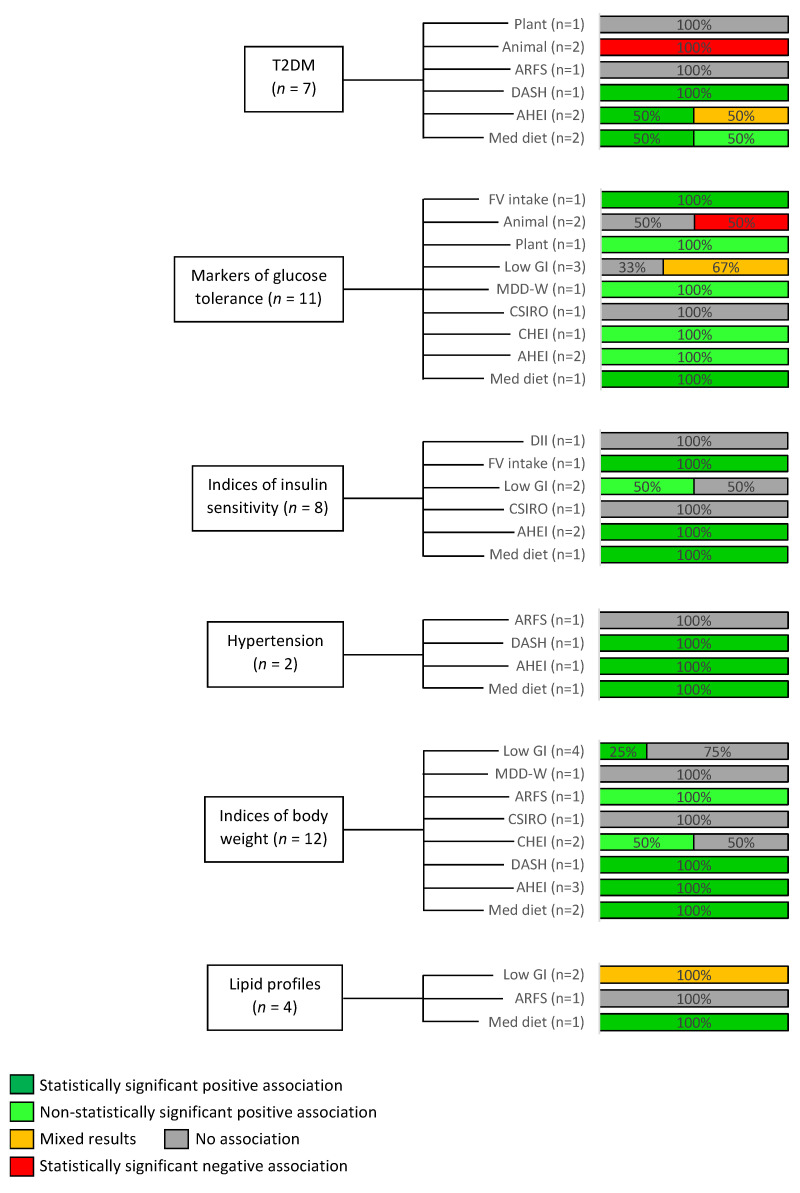
Overview of outcomes reported within included studies and the proportion of studies evaluating these outcomes which demonstrated an association with specified dietary patterns. Markers of glucose tolerance’ includes fasting plasma glucose, 2-h post-glucose load glucose levels, and HbA1c which were variously reported and combined to allow for the determination of overall patterns. Similarly, ‘indices of insulin sensitivity’ includes Matsuda index and HOMA-IR. ‘Indices of body weight’ including BMI, body fat, and waist/hip ratio were similarly clustered, and variously reported lipid parameters were also collated to allow comparison. A positive association refers to a desired outcome i.e., improved glucose tolerance and reduced incidence of hypertension. Plant: low-carbohydrate diet with higher proportion of macronutrients from plant sources, Animal: low-carbohydrate diet with higher proportion of macronutrients from animal sources, AHEI: Alternate Healthy Eating Index, Med Diet: Mediterranean Diet, DASH: Dietary Approaches to Stop Hypertension, Chinese Healthy Eating Index, CSIRO: Commonwealth Scientific and Industrial Research Organisation, ARFS: Australian Recommended Food Score, MDD-W: Minimum Dietary Diversity for Women, GI: Glycaemic Index, DII: Dietary Inflammatory Index.

The included studies varied widely in their methodologies and outcomes of interest. Figure 2 gives an overview of the outcomes reported within the included studies and the dietary patterns which were explored in relation to these outcomes.

### 3.4. Risk of Incident T2DM

Of particular interest is the role of dietary patterns in the development of T2DM in women with GDM. Two studies evaluated the association between the Mediterranean dietary pattern on T2DM incidence. The randomised controlled study by Perez-Ferre et al. demonstrated that their intervention promoting the Mediterranean dietary pattern was associated with a reduction in the rate of conversion to a glucose disorder, and that the intervention group had a higher adherence to the MD pattern than the control group. However, the effect on the rates of incident T2DM did not reach statistical significance [65]. Tobias et al. evaluated the aMED scores for participants in the Nurses’ Health Study T2DM [61]. This scoring system was derived from the Mediterranean diet and adapted for a United States population [67]. They demonstrated that increased aMED scores were associated with a reduced risk of T2DM. The study by Tobias et al. also evaluated the correlation of both the DASH diet and AHEI on the development of T2DM. For both DASH and AHEI, in addition to MD, adherence was found to be inversely correlated with incident T2DM [61]. They reported that the correlation was strongest with AHEI, with a 57% reduction in risk between the highest adherence quartile and the lowest (HR = 0.43 [95% CI: 0.31, 0.59] *p*-trend < 0.0001), compared to 40% (HR = 0.60 [95% CI: 0.44, 0.82] *p*-trend = 0.0019) and 46% (HR = 0.54 [95% CI: 0.39, 0.73] *p*-trend = 0.0002) lower risk for MD and DASH, respectively. A study by Li et al. aimed to examine the association of a genetic risk score with risk of T2DM in women with a history of GDM, and how the association might be modified by non-genetic determinants for T2DM [68]. One non-genetic determinant that was included was AHEI adherence. Two cohorts were studied: the NHSII cohort which was previously reported on in the above study by Tobias et al. [61] and the Danish National Birth Cohort (DNBC). As above, they reported that for the NHSII cohort, the women who developed T2DM had a lower AHEI score than those who did not develop T2DM. However, for the DNBC there was no significant difference in the AHEI score. One study evaluated the association between adherence to ARFS and T2DM incidence: whilst it was not a stated aim of the study, the cross-sectional study of 1447 women by Morrison et al. did not find any differences in the T2DM incidence among women with higher adherences to the ARFS [64].

Of the five studies which reported on incident T2DM, two evaluated the effect of plant versus animal dietary components. Both were cohort studies. In their analysis of the Nurses’ Health Study, Bao et al. reported that low-carbohydrate diets (LCDs) with preferential substitution of carbohydrates with fat and protein from animal sources were significantly positively associated with the development of T2DM in women with a history of GDM [62]. The multivariable-adjusted HRs (95% Cis) of T2DM comparing the highest with lowest quintiles were 2.13 (1.65–2.76) for the overall LCD score (*p* < 0.001 for trend) and 2.18 (1.68–2.83) for the animal LCD score (*p* < 0.001 for trend). However, they report that low-carbohydrate diets with preferential substitutions with fat and protein from plant sources were not associated with increased T2DM risk. Kim et al. also reported that participants in their cohort study with higher energy and protein and fat intake from animal sources had higher rates of T2DM, with women with T2DM consuming almost twice as much of their recommended total intake of protein and fat from animal sources as women with normal glucose tolerances [63]. 

As can be seen in Figure 2, no studies evaluated the relationship between adherence to a low GI diet and the subsequent development of T2DM.

### 3.5. Markers of Glucose Tolerance

Several studies reported markers of glucose tolerance such as fasting plasma glucose, 2-h post-glucose challenge glucose levels, and HbA1c. Whilst no studies reported on the effect of the low GI diet on T2DM incidence, three reported on other markers of glucose tolerance. In the RCT by Shyam et al., a low GI dietary intervention resulted in statistically significantly lower GI diet scores at 6 months, compared to the control group who received conventional healthy dietary recommendations (CHDRs). They reported statistically significant reductions in 2-h post-glucose challenge glucose levels in the low GI group compared to conventional advice at 6 months (low GI vs. CHDR: median (IQR): −0.2(2.8) vs. +0.8 (2.0) mmol/L, *p* = 0.025) [69]. However, they found no differences in the fasting glucose levels. In the follow-up study of the same cohort at one year, Ghani et al. reported statistically significant improvements in fasting plasma glucose compared to the those who received CHDR at one year, but only for women who had higher baseline fasting insulin levels (indicating a degree of persisting insulin resistance): the low GI group had a 2.2% reduction from the baseline in FBG (−0.12 ± 0.27 mmol l^−^^1^ ), whereas CHDR subjects had a 3.8% increase (0.17 ± 0.32 mmol l^−^^1^) (*p* = 0.025) [70]. No such improvements were seen in women who had lower fasting insulin (and, therefore, were deemed to be more insulin sensitive at the baseline). Louie et al. carried out a follow-up study of a RCT whereby women with a GDM pregnancy were randomised to either a low GI diet or a conventional high-fibre diet at 29 weeks’ gestation [71]. The intervention stopped at 36–37 weeks’ gestation, but participants were encouraged to adhere to their assigned diet in the post-partum period. In total, 58 women returned at 3 months postpartum to take part in the study by Louie et al., with 73% of participants deemed to be still compliant with the study diet; they found that the low GI education intervention had no effect on fasting or the 2-h glucose levels in the post-partum period. This was a follow-up study with a reduced cohort and was underpowered to detect any statistically significant differences in glycaemic parameters. 

Several studies reported on the effects of additional ‘healthy’ dietary patterns on abnormal glucose tolerance. The study by Perez-Ferre, in addition to reporting on T2DM incidence, found non-statistically significant trends towards a reduction in FPG and rates of glucose intolerance in the MD group compared to the control group [65]. A retrospective cohort study by Gingras et al. evaluated the effect of the adoption of up to three ‘preventative practices’ on anthropometric and metabolic profiles in women with a history of GDM [72]. One of the preventative practices was adherence to AHEI and, whilst the reduction in risk associated with high adherence to AHEI alone was not reported, they did provide adjusted Pearson’s correlation coefficients between the AHEI score and metabolic parameters. No changes in FPG were reported with the increased adherence to AHEI and there was a non-significant trend towards a reduction in 2 h glucose. In addition, examining the role of AHEI, Ferranti et al. carried out a cross-sectional descriptive study of 75 women with a history of GDM over a mean follow-up period of 2.6 years. They reported that better AHEI adherence was protective for HbA1c (*r* = −0.23, *p* = 0.02) and the risk of metabolic syndrome (*t* = 2.2, *p* = 0.03) [73].

One study was found which evaluated the role of the CHEI. The randomised controlled trial aimed to evaluate the effect of intensive lifestyle modifications in two centres within rural areas of Hunan Province, China, on physiological health outcomes including the development of T2DM and weight-related variables, and was reported over several articles by the group [56]. After the 6-month intervention, there were significant reductions in fasting blood glucose, 2-h glucose at OGTT. and T2DM risk score in the intervention group compared to the control group (*p* < 0.05) [57]. Whilst CHEI adherence was not directly reported in this article, an additional article from the group reported on CHEI adherence at 18 months within the study. Whilst this was not directly correlated with metabolic outcomes, they reported that after 18 months the intervention group achieved a higher CHEI score than the control group, having been equivalent at the baseline [55]. They also reported that, in a generalised linear mixed model, the lifestyle intervention significantly increased the total CHEI score (*p* = 0.049) after other factors were included in the analysis.

In the above article by Li et al. describing the changes in CHEI adherence 18 months following the intervention, they also reported on the proportion of participants reaching the MDD-W [55]. At the baseline, the proportion of individuals reaching the MDD-W was similar in the intervention and control groups and after 18 months, there were significantly more women achieving the MDD-W in the intervention group compared to the control group (90.6% vs. 81.2%, *p* = 0.023) [55]. However, the lifestyle intervention was not significantly associated with MDD-W status. Therefore, whilst the intervention resulted in improved metabolic outcomes [57], the relationship between MDD-W and glucose tolerance in this population is unclear.

An article by Gray et al. reported the CSIRO-HDSs as part of an exploratory secondary analysis of a 12-month trial comparing intermittent energy restriction (IER) to continuous energy restriction (CER) following GDM [58]. In this secondary exploratory analysis, they reported that whilst the CSIRO-HDSs improved in both groups between the baseline and 12 months, the CER group showed a weak statistically significant improvement compared with the IER group. They did not correlate the CSIRO-HDS with any metabolic outcomes in this study. However, the results of the original trial reported on weight loss, HbA1c, fasting plasma glucose, fasting serum insulin, HOMA-IR, and 2-h oral glucose tolerance at 12 months in each group [59]. They reported that there are no differences in these outcomes between the IER and CER groups. In the original study, there was a 49% attrition rate which limits interpretation and, with a further reduction in numbers due to incomplete dietary quality reporting, it is not clear whether adherence to the CSIRO-HDS is correlated with weight or metabolic outcomes in women with a history of GDM.

Two studies evaluated the effects of predominantly animal-based low-carbohydrate diets on markers of glucose tolerance. In the previously mentioned study by Kim et al., it was reported that higher animal fat and protein consumption as part of a low-carbohydrate diet was associated with increased rates of prediabetes [63]. Tang et al. carried out a prospective cohort study aiming to evaluate the association between LCDs and the risk of IFG at 6–8 weeks postpartum in women with and without GDM [74]. While overall they reported a trend towards a positive association between the overall LCD score and FPG and a negative association between plant LCD score and FPG, they found no correlation between the animal/plant LCD scores and fasting plasma glucose in women with GDM. Mercier et al. carried out an analysis of the women with GDM in the cohort study conducted by Gingras et al. reported above, to look specifically at fruit and vegetable intake and the association with the subsequent development of abnormal glucose tolerance (AGT). In a cross-sectional analysis at 5.9 + 3.0 years postpartum, it was reported that women with AGT had significantly lower fruit/vegetable servings than women with NGT (6.5 ± 0.2 vs. 7.4 ± 0.2, *p* = 0.001) [75]. The vegetable intake alone was also significantly lower in those with AGT (3.9 ± 0.2 vs. 4.5 ± 0.2, 0 = 0.04), and there was a trend towards reduced fruit intake. The authors also reported that per one serving size increase, the fruit/vegetable intake was associated with a reduced likelihood of having AGT (OR = 0.88 (0.81–0.97)) after adjusting for age and BMI.

### 3.6. Indices of Insulin Sensitivity

As part of their analysis, Perez-Ferre et al. reported that the group adhering to a MD had lower fasting insulin levels and lower HOMA-IR than the control group [65]. Gingras et al. reported that, in their cohort, AHEI adherence was significantly correlated with reduced fasting insulinaemia, 2-h post-OGTT insulinaemia, and a higher Matsuda index [72]. Ferranti et al. reported that better AHEI adherence was correlated with a lower risk of metabolic syndrome [73]. The study by Gray et al. reported no significant differences in fasting insulin or HOMA-IR between groups with significantly different CSIRO-HDSs, although again it should be noted that the study was not powered to detect such changes [58].

The studies by Louie et al. and Shyam et al. reported on the markers of insulin sensitivity in relation to low GI diets. In their post-natal follow-up study, Louie et al. found no significant differences in fasting insulin or HOMA-IR between the group assigned to adhere to a low GI diet and those assigned to a conventional high-fibre diet during pregnancy. While there were no statistically significant differences, they commented that the small reductions observed in these parameters were likely to be of clinical significance (fasting insulin 39.6 (32.8–46.3) in the low GI group, 53.8 (35.6–71.9) in the HF group) [71]. Seventy-three percent of women were deemed to still be adherent to their assigned diet during the post-natal period, but metabolic parameters were not evaluated in these women alone. Shyam et al. reported no significant differences in fasting insulin between the groups in their study evaluating the effect of a low GI intervention compared to the CHDR control group [69].

In the cohort study by Mercier et al., they reported that, in women with abnormal glucose tolerance, vegetable intake was negatively correlated with HOMA-IR and positively correlated with the Matsuda index, which correlates with the previously described effects on glucose tolerance [75]. None of the other studies evaluating the role of plant- or animal-based dietary components reported on indices of insulin sensitivity.

Shin et al. utilised data from the National Health and Nutrition Examination Survey to evaluate insulin resistance, as estimated by the HOMA-IR, in a cohort of 2674 women [76]. While they found that in women without a history of GDM, those in the highest tertile of DII scores had an increased risk of insulin resistance compared to those in the lowest tertile, no such associations were found in women with a history of GDM (*n* = 176). This may suggest that a lower DII does not reduce the risk of insulin resistance in women with a history of GDM. However, the data are presented in an abstract form and there is no information supplied regarding the outcomes to which the study was powered. It is likely that examining the effect in women with a history of GDM was not the primary outcome of interest and that it was, therefore, underpowered to detect these changes.

### 3.7. Modifiable Cardiovascular Risk Factors

No studies evaluated the relationship between dietary patterns and the risk of incident cardiovascular disease in women with GDM. However, several studies evaluated the associations with modifiable cardiovascular risk factors. The study utilising the Nurses’ Health Study cohort by Li et al. found that the incidence of hypertension was reduced in those with a higher adherence to the Mediterranean diet, the AHEI, and the DASH diets [77]. The hazard ratios and 95% confidence intervals comparing the highest and lowest quartiles were 0.76 (0.61–0.94; *p* for linear trend = 0.03) for AHEI score, 0.72 (0.58–0.90; *p* for trend = 0.01) for DASH score, and 0.70 (0.56–0.88; *p* for trend = 0.002) for aMED. Morrison et al. found no correlation between quintiles of ARFS adherence and incidence of hypertension in their cohort study [64]. However, the study was not powered to detect these changes.

The study by Franzago et al. demonstrated a non-significant correlation between the PREDIMED scores (an MD score) and cIMT values [66]. It should be noted that this was a secondary analysis, and the study was not powered to detect any correlation between diet scores and cIMT. cIMT is a tool which is used to assess the cumulative effect of atherosclerotic risk factors and is an independent risk factor for cardiovascular disease [78]. However, the clinical utility of cIMT as a component of cardiovascular risk prediction is uncertain. 

Several studies evaluated the effect of dietary pattern adherence on weight or markers of body fat. In the RCT by Perez-Ferre, they found that the group in the Mediterranean diet intervention had lower waist circumferences and BMIs than the control group [65]. Tobias et al. similarly found that a higher adherence to the Mediterranean diet was associated with a lower body weight in their cohort study [30]. They also found that a higher adherence to both the DASH and AHEI diets was associated with a lower body weight. Two other studies evaluated the effect of AHEI adherence on markers of body weight. The retrospective cohort study by Gingras et al. reported that AHEI adherence was significantly negatively correlated with waist circumference and body fat percentage and Ferranti et al. reported that better AHEI was protective for BMI [72,73]. The AHEI adherence was also correlated with BMI, but this was not statistically significant. In the study by Morrison et al. there was a trend such that women with a higher BMI were less likely to be in the highest compared to the lowest ARFS quintile, but this did not reach significance [64]. In the study by Gray et al., there were no differences in body weight between groups in their exploratory analysis of CSIRO-HDSs in IER and CER groups [58].

Four studies commented on the relationship between low GI adherence and markers of body weight. Shyam et al. found that the low GI group in their study had lower bodyweights, BMIs, waist circumferences, and waist:hip ratios at 6 months [69]. However, in the one-year follow-up study of the same cohort, Ghani et al. found no significant differences in body weight, body fat, or trunk fat between groups [70]. They did report that there was a trend towards more weight loss in women with higher fasting insulin levels at the beginning of the study who followed low GI recommendations compared to the control group; however, this was not statistically significant. In their cross-sectional study, Ott et al. reported no significant correlation between the dietary glycaemic load and post-partum BMI [79]. However, this was not a prior stated aim of their study. The cohort study by Nicklas et al. found no association between dietary glycaemic load and early weight loss in women with recent GDM [80]. The numbers in the study were small and the aim of the study was to determine whether there were any factors (dietary and otherwise) which were associated with early weight loss.

Two studies reported on the relationship between CHEI adherence and markers of body weight. In the study of dietary quality among women with previous GDM by Li et al., it was reported that CHEI scores were low in this population, with no difference across the BMI categories [56]. Chen et al. reported that participants in the lifestyle intervention group had higher reductions in waist circumferences compared to those in the control group at 6 months. As highlighted earlier, an additional article from the group reported that after 18 months the intervention group achieved a higher CHEI score than the control group [55]. It could therefore be inferred that there is an association between CHEI adherence and reduced waist circumference. This article also reported that a higher proportion of the intervention group achieved MDD-W compared to the control group but that the intervention was not associated with MDD-W achievement in a generalised linear mixed model. The association between MDD-W and waist circumference is, therefore, unclear. 

Lipid profiles were variously reported across the studies included within this review. Perez-Ferre reported that, in their study, those assigned to the Mediterranean diet intervention had significantly lower triglycerides, apolipoprotein B, and LDL cholesterol than the control group [65]. Morrison et al. reported no difference in the incidence of hyperlipidaemia between ARFS quintiles [64]. The analyses by Ghani et al. and Louie et al. reported on the effect of low GI dietary adherence and lipid profiles. In the RCT by Ghani et al., there were no differences in HDL, LDL, total cholesterol:HDL ratio, or LDL:HDL ratio between groups, but they did report significantly lower triglycerides in the low GI group [70]. In the post-natal follow-up study by Louie et al., they found a non-significant reduction in triglycerides in the group which had been assigned to a low GI diet compared to the control group [71]. They did not find any significant differences in HDL, LDL, total cholesterol, or free fatty acids. They did not stratify these findings by post-natal adherence to the assigned dietary pattern and so the relationship between post-natal low GI adherence and lipid profiles in this cohort is unclear.

## 4. Discussion

This scoping review summarises the literature surrounding the relationship between dietary patterns and cardiometabolic risk in women with a history of GDM. Twenty-four studies met the inclusion criteria. Women with a history of GDM are up to 10 times more likely to develop T2DM later in life than those without GDM [3]. For this reason, much research activity has focussed on reducing the risk of progression to T2DM in this population, with interventions primarily targeting diet and physical activity. Where the specific dietary advice given is detailed within studies, it is often based around recommendations for those at risk of T2DM, for example, following the advice within the Diabetes Prevention Programme (DPP). The DPP endorses a low-fat, high-fibre diet low in processed foods, and is endorsed by the National Institute for Health and Care Excellence (NICE) [81]. The evidence considered in formulating these recommendations concentrates on the populations at higher risk of developing T2DM, including those with obesity, hypertension, raised fasting plasma glucose, and a family history of T2DM. In these populations, studies evaluating the efficacy of DPPs report a reduced T2DM incidence, weight, and HbA1c [82]. Whilst a history of GDM is included within the definition of high risk in these studies, there is a paucity of studies exploring the optimal recommendations specifically for those with a history of GDM. Indeed, in a 10-year follow-up study of the DPP in women with and without a history of GDM, women with a history of GDM following the DPP lost less weight than women with no GDM history [83]. Intensive lifestyle intervention in the cohort with GDM still resulted in a 35% reduction in T2DM incidence. However, the modest effects on weight loss compared to those with no GDM suggests that a tailored approach for women with GDM may be necessary. The National Institute for Health and Care Excellence has established that further study is urgently needed to assess the effectiveness of post-partum weight management interventions to reduce the risk of T2DM in women with GDM [84].

The primary aim of this review was to identify whether any specific dietary patterns are associated with a reduction in risk of T2DM and CVD in women with a history of GDM. Five studies were identified which reported on the risk of T2DM: one observational study reporting on MD, DASH, and AHEI adherence; one RCT of an intervention encouraging adherence to MD; two cohort studies reporting on the relative consumption of plant/animal products; and one cross-sectional study reporting on ARFS adherence (although this was not a pre-defined aim of the study). Tobias and colleagues concluded that higher adherence to the MD, DASH diet, or AHEI is associated with a lower risk of T2DM, the correlation being strongest with AHEI [61]. No direct comparisons between the dietary patterns were performed within the study. The authors comment that they used previously published scoring methods for each dietary pattern, producing significantly different scales, and that any differences between dietary patterns may, therefore be explainable by the precision of the exposure measurements. Furthermore, the three dietary patterns share many common components so it is therefore challenging to decipher whether there are any clinically relevant differences. It is possible that the substitution of potentially harmful dietary components such as red meat for alternative protein sources influenced the risk of T2DM. This would align with the aforementioned observations that the higher ingestion of fat and protein from animal sources is associated with increased T2DM risk. The three dietary patterns explored within this study are also associated with higher intakes of complex carbohydrates, which may slow intestinal glucose absorption and reduce beta cell demand.

Another healthy dietary pattern, the ARFS, was not reported to be associated with T2DM risk, although detecting these changes was not a stated aim of the study. The aim of the study was to describe the diet quality of a sample of women with recent GDM and to determine any factors associated with adherence to national dietary guidelines. It was not powered to detect changes in chronic disease incidence and, therefore, this study does not necessarily demonstrate that ARFS adherence is not related to T2DM incidence in this population. Furthermore, it is also not clear what the mean time from GDM diagnosis to survey was and so it is possible that any post-natal dietary alterations were of insufficient durations as to have had any effect. Additionally, the ARFS possesses similar components to the other healthy dietary patterns described and it is likely that a benefit would be conveyed due to these underlying similarities.

Only one RCT was identified where the intervention encouraged, and then adherence measured, a specified healthy dietary pattern. The study by Perez-Ferre et al. found that the participants who were in the intervention arm promoting adherence to MD had lower rates of conversion to glucose disorders than those in the control arm at 3 years; however, there were no statistically significant effects on the risk of T2DM [65]. There was a trend towards a reduction, and they report statistically significant improvements in body weight and lipid profiles. It is possible that the sample size was insufficient to detect differences in incident T2DM, having been powered only to detect changes in the rates of glucose disorders overall. The period of time between a pregnancy affected by GDM and subsequent T2DM diagnosis can be prohibitively long for RCTs, and this may explain the lack of RCTs evaluating the effect on T2DM risk and the relative prevalence of observational studies. Further study is required to determine the optimal dietary patterns to include within post-partum lifestyle interventions for women with T2DM to reduce the risk of subsequent T2DM. This study was one of two studies evaluating the relationship between MD adherence and T2DM, and there were no others reporting whether there are differences in biochemical indicators of insulin resistance or glucose intolerance. It is, therefore, difficult to draw conclusions about the effect of MD on the development of T2DM in women with GDM so further study is needed. Similarly, the limited number of studies evaluating the association of other healthy dietary patterns and subsequent risk of T2DM preclude the formulation of any strong conclusions regarding potential benefit.

Two studies evaluated the associated plant versus animal macronutrients as part of a low-carbohydrate diet. Bao et al. reported that low-carbohydrate diets with preferential substitutions of carbohydrates with fat and protein from animal sources were significantly positively associated with the development of T2DM but that low-carbohydrate diets with preferential substitutions with fat and protein from plant sources were not associated with increased T2DM risk [62]. Kim et al. also reported that participants in their cohort study with higher energy and protein and fat intake from animal sources had higher rates of T2DM and prediabetes [63], suggesting that low-carbohydrate diets with high consumptions of animal products may increase the risk of incident T2DM in women with GDM. These results contrast with another study of the same cohort used by Bao et al., which looked at all women, not just those with a history of GDM. Halton et al. created an LCD score using the percentage of energy as carbohydrate, the percentage of energy as animal protein, and the percentage of energy as animal fat [85]. In this expanded cohort, there was no increased risk of development of T2DM. However, a higher LCD score using the percentage of energy as carbohydrate, percentage of energy as vegetable protein, and percentage of energy as vegetable fat was associated with a reduced risk of the development of T2DM, with a relative risk of 0.82 comparing the 10th with the 1st decile for this score. Whilst there is significant variation in the results of studies evaluating the effect of the proportion of plant versus animal dietary components, a general pattern emerges which suggests that LCDs, in particular LCDs with a high proportion of protein and fat originating from animal sources, may be associated with the development of T2DM, particularly in women with GDM. Further study is required to investigate the role of plant/animal LCDs in the development of T2DM in women with a history of GDM.

Within the current review, the low GI diet was a commonly described diet, featuring in five articles. However, none of these reported T2DM incidence as an outcome. The current dietary advice given to women with GDM and patients with T2DM promotes the incorporation of high-fibre, low GI sources of carbohydrate into the diet, in place of high GI refined carbohydrates [84,86]. It is, therefore, perhaps surprising that no studies sought to evaluate the effect of the low GI diet on subsequent T2DM incidence, and this represents a current gap in the literature.

No studies reported on the incidence of cardiovascular disease in relation to the dietary pattern. A recent meta-analysis demonstrated that women with a history of GDM are twice as likely to develop cardiovascular disease later in life [4]. It is well established that adherence to healthy dietary patterns reduces the risk of cardiovascular disease in at-risk populations, but this review did not find any evidence of the specific study of women with GDM. The absence of evidence regarding the association between healthy dietary patterns and cardiovascular disease in this population is, therefore, a significant gap in the literature.

Several articles described the relationship between dietary pattern adherence and subsequent markers of glucose tolerance and insulin sensitivity. Shyam et al. reported that their low GI intervention improved the 2-h post-glucose load levels at 6 months, but these effects were not evident at 1 year [69]. In the same cohort, Ghani et al. reported statistically significant improvements in fasting plasma glucose for women who had higher baseline fasting insulin levels but not in women who had lower fasting insulin levels [70]. This suggests that the low GI diet might be more beneficial in reducing the risk of impaired fasting glucose in women who have higher levels of insulin resistance. There were no differences in the indices of insulin sensitivity in this cohort at 6 months and they were not reported at 1 year. Louie et al. reported no statistically significant changes in the markers of glucose tolerance or insulin sensitivity in their low GI intervention study but they did comment that the small reductions in fasting insulin and HOMA-IR seen were likely to be of clinical significance [71]. This was a follow-up study which was underpowered to detect any statistically significant differences in glycaemic parameters. Furthermore, around a quarter of participants were no longer adhering to their diet and the metabolic parameters were not separately evaluated in those participants who were still adherent. Given the conflicting evidence in these small studies regarding the role of low GI in mediating glucose tolerance following GDM, further study is required.

The effect of adherence to healthy dietary patterns on glucose tolerance and insulin sensitivity was also reported. Whilst the findings for individual markers varied, there is a general trend towards an increased adherence to healthy dietary patterns being associated with increased insulin sensitivity and glucose tolerance. The overall number of articles describing these markers was small (five articles describing four healthy dietary patterns) and further study is warranted. One study evaluating a lifestyle intervention, reported over several articles, found significant reductions in 2 h and fasting glucose in the group assigned to the lifestyle intervention which also achieved higher CHEI scores. This suggests that higher adherence to CHEI may improve metabolic outcomes in women with GDM, although it is worth noting that the dietary advice was only one component of the intervention, with physical activity also targeted. There were, however, no reported differences in the physical activity levels between groups at 6 months [57]. The results from the ongoing trial may provide useful insights into the correlation between CHEI adherence and glycaemic health and cardiometabolic risk factors. For example, post-partum RCTs in women with recent GDM often have a dietary component as part of a broader lifestyle intervention which involves increased physical activity. It can therefore be difficult to ascertain the role that the dietary patterns may play in isolation.

Several risk factors for cardiovascular disease and T2DM were reported on within the studies included in the review. A raised BMI is a significant risk factor for both T2DM and cardiovascular disease and weight-related indices were therefore predictably a prominent primary outcome in studies within this review. Adherence to the more commonly studied dietary patterns (MD, AHEI, and DASH) was most consistently associated with a reduction in weight-related indices. As discussed above, there are significant similarities between these dietary patterns and the effects on weight are possibly mediated via the same underlying mechanisms. The articles addressing the impact of a low GI diet on outcomes for women with GDM provided less convincing evidence of an effect on body weight. Shyam et al. reported a lower body weight, BMI, and waist circumference in their low GI intervention group than in the control group at 6 months. However, there was no effect in the one-year follow-up study by Ghani et al. The other studies which reported no effect were small and did not have differences in anthropometric measurements as a primary outcome. They were, therefore, unlikely to detect any significant differences between groups. Weight loss in the post-partum period is associated with a reduced risk of T2DM [87]. Indeed, in the study by Tobias et al., BMI was estimated to mediate 50%, 41%, and 39% of the effect of AHEI, MD, and DASH adherence, respectively, on the development of T2DM. Further research is required to determine whether any specific dietary patterns are associated with improvements in anthropometric measures, which may subsequently mediate improvements in metabolic health.

Additional modifiable risk factors which were reported included the lipid profiles. The MD RCT by Perez-Ferre reported improved lipid profiles, and the study evaluating adherence to the ARFS found no differences in the lipid profiles. There would appear to be an association between adherence to a low GI diet and a reduction in triglycerides in women with a history of GDM, but further study is required. This would be consistent with other studies in the literature which demonstrate an association between low GI adherence and lower triglycerides [88]. Hypertension was evaluated in two studies. Li et al. found that adherence to AHEI, MD, and DASH reduced the incidence of hypertension, and no association was found in the small cohort study evaluating the effect of adherence to the ARFS. One study reported on cIMT and found a non-significant correlation between Mediterranean diet scores and cIMT values. This was a secondary analysis and, as it was the only study in this category, further study is required to draw any conclusions on the relationship between healthy dietary pattern adherence and cIMT.

**Figure 3:**
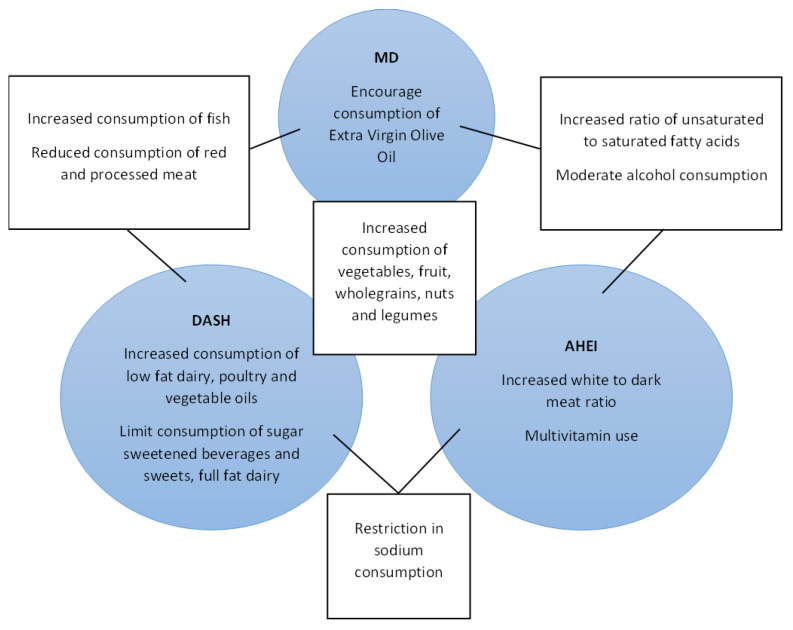
Dietary components of the **MD**, **DASH**, and **AHEI**, arranged to depict the similarities between the dietary patterns.

Considering the dietary patterns identified within this review, four distinct groups were identified: those considered to be ‘healthy’ dietary patterns (the largest group), those focussing on the glycaemic index, those centred around plant- versus animal-based dietary components, and one pattern quantifying the diet’s inflammatory potential. Whether the specific patterns studied are ideal in terms of components related to the health outcome is uncertain. Six identified patterns are considered to be healthy dietary patterns which either contain similar components or have been adapted to improve the representation of culturally appropriate foods. The results of this scoping review have highlighted that healthy dietary patterns are, on the whole, associated with positive metabolic outcomes in women with a history of GDM. The large number of healthy dietary patterns makes the direct comparison of study outcomes difficult; however, there are several dietary components that are common to all of these dietary patterns. Figure 3 depicts the common features of the three most well-described ‘healthy’ dietary patterns within this review. It can be seen that the consumption of vegetables, fruits, wholegrains, nuts, and legumes are encouraged in all three diets. Some differences exist between the patterns; however, these do not necessarily reflect disparate dietary components in practical terms. For example, MD and DASH encourage reduced consumption of red and processed meats, whilst for AHEI the ratio of white to dark meat consumption is scored. The DASH and MD recommend Increased consumption of fish and DASH encourages poultry consumption. Practically, these descriptors are likely to reflect similar patterns of animal protein consumption. It is probable that the similarities between these healthy dietary patterns mediate the reduction in cardiometabolic risk and so the benefit of recommending one approach over another is uncertain. The lack of knowledge regarding the specific dietary advice that should be followed has been cited as a barrier to dietary modification in the post-partum period in women with a history of GDM [89]. Time restraints are also frequently cited as a barrier in this population [90]. The simplification of dietary advice which can be readily adapted to individual circumstances could make any proposed intervention easier to implement and improve engagement.

### 4.1. Strengths and Limitations

One strength of this review is its broad scope, aiming to explore the breadth of the literature in the area. This has enabled the presentation of an extensive overview of the evidence and identification of gaps in the current literature. Many of the articles reported on outcomes of interest to this review that were not specified outcomes of the original studies and were, therefore, not powered as such. This is a limitation of the current review as it may have resulted in the presentation of null findings where real effects exist. However, the inclusion of these articles also represents a strength of the review as it allows full exploration of the published literature. An additional limitation of this review is that some included studies reported on the outcomes of interest but not the adherence to dietary patterns [55]. Where other publications from the same group of the same cohort presented dietary information, inferences were made about the relationship between the dietary pattern presented in one article and the outcome in another. Whilst the included populations may have differed slightly, the cohorts were the same and so it was felt relevant to include these studies in the interest of exploring the full extent of the literature. A formal quality assessment of included articles is beyond the scope of a scoping review and this is recognised as a limitation. Additionally, the inclusion of all types of quantitative study designs makes the direct comparison of included studies challenging. The articles included within this review were either trials of a described intervention or observational cohort studies. Cohort studies requiring participant reporting of dietary information are subject to recall and response bias. Furthermore, the potential for confounding limits the strength of evidence regarding reported outcomes. Additionally, due to the duration of cohort studies, they are particularly susceptible to attrition. Over prolonged periods there is the potential for changes to the diagnostic criteria (as has occurred with GDM), and so it is also possible that the cohort being observed is changing with time. The cohort studies within this review that evaluated the impact of healthy dietary patterns suggest that there is an improvement in cardiometabolic outcomes with increased adherence; this has been supported by some RCTs also included within this review. 

The formal literature search was conducted in March 2022 and it became evident to the authors that some additional articles may have been published which were of relevance prior to the completion of the manuscript. An additional limited search of PubMed was, therefore, carried out to ensure the capturing of these articles. An additional formal literature search may have been preferable, but it was felt that the short time period in question was not likely to yield significant numbers of relevant articles.

This scoping review has provided a broad view of the literature surrounding the relationship between dietary patterns and cardiometabolic health in women with a history of GDM. Given the volume of published post-natal interventions for women with GDM which include a dietary component GDM [23,24], the number of studies which have sought to determine the dietary approaches which could be most beneficial are limited. During the formal literature search, additional research protocols were identified which will likely further add to the evidence regarding the role of dietary patterns in mediating cardiometabolic risk in women with a history of GDM. A protocol by Bolou et al. describes a feasibility study which will assess the acceptability of a MD intervention from 6–13 weeks post-partum until one-year post-partum in women with recent GDM [91]. They aim to evaluate adherence to the dietary intervention as well as rates of maternal dysglycaemia. In addition, an ongoing trial which has been reported in this review will also likely yield further useful information regarding the relationship between the CHEI and metabolic outcomes [55,56,57]. 

### 4.2. Future Work

A large proportion of the evidence presented within this review focuses on healthy dietary patterns, primarily derived from observational studies. These studies suggest that healthy dietary patterns might reduce cardiometabolic risk. However, RCTs of interventions promoting adherence to healthy dietary patterns, with sufficient power to determine whether there is any effect on the incidence of T2DM, are required. A number of significant gaps in the literature were identified which also warrant further study. Low GI diets are considered prudent advice for women with GDM [84]. However, no studies were identified which evaluated its association with subsequent development of T2DM in this cohort. Further study is required in this area to determine whether the current dietary advice will be of metabolic benefit in the longer term. Finally, no studies evaluated the association between dietary patterns and incident CVD. Women with a history of GDM are twice as likely to develop CVD as their normoglycaemic counterparts [4], and further research is required to evaluate whether specific dietary patterns can reduce this risk, as has been demonstrated in the general population [36,37,41]. If specific dietary patterns were identified which reliably reduced T2DM and CVD risk and/or improved modifiable cardiometabolic risk factors in women with a history of GDM, it would allow for a tailored approach to risk reduction interventions in this cohort, optimising primary prevention and reducing morbidity secondary to cardiometabolic disease.

## Data Availability

Data described in the manuscript can be shared publicly upon reasonable justified request.

## Supplementary Materials

The following supporting information can be downloaded at: https://www.mdpi.com/article/10.3390/nu15071613/s1, Table S1: Overview of the characteristics, aims, and main findings of included studies.

## Appendix A. Search Terms Employed for Literature Searches

### Appendix A.1. EMBASE and Medline Search Terms

**NUMBER**	**SEARCH TERM**
1	Diabetes, Gestational
2	low fiber diet/or Mediterranean diet/or high-fructose diet/or lactovegetarian diet/or modified Atkins diet/or diet composition/ or vegan diet/or “high fat/high fructose diet”/or Nordic diet/or healthy diet/or cariogenic diet/or ovovegetarian diet/or lipid diet/or high-glucose diet/or lactoovovegetarian diet/or high fiber diet/or diet supplementation/or low fat diet/or protein diet/or diet therapy/or six food elimination diet/or diabetic diet/or raw food diet/or low FODMAP diet/or low calorie diet/or fruitarian diet/or very low calorie diet/or lithogenic diet/or high glycemic index diet/or atherogenic diet/or elemental diet/or gluten free diet/or gluten free casein free diet/or very low calorie ketogenic diet/or high-protein low-carbohydrate diet/or “high fat/high glucose diet”/or casein free diet/or low glycemic index diet/or obesogenic diet/or “high fat/high sucrose diet”/or pescovegetarian diet/or lactose free diet/or low iodine diet/or MIND diet/or low residue diet/or carbohydrate diet/or Atkins diet/or low carbohydrate diet/or liquid diet/or paleolithic diet/or Western diet/or experimental diet/or high methionine diet/or macrobiotic diet/or fiber free diet/or ketogenic diet/or high calorie diet/or soft diet/or high salt diet/or cafeteria diet/or artificial diet/or cereal-based diet/or high-sucrose diet/or elimination diet/or diet/or renal diet/or cholesterol diet/or full liquid diet/or diet restriction/or clear liquid diet/or vegetarian diet/or diet-induced obesity/or “methionine/choline deficient diet”/or unhealthy diet/or carbohydrate loading diet/or DASH diet/
3	“diet”
4	Diet*
5	Low fibre diet.mp
6	“Dietary pattern”
7	Dietary pattern *.mp
8	Dietary habit
9	“Eating habit”
10	Dietary habit *.mp
11	Nutrient pattern*.mp
12	Diet, high-fat/or diet, western/or diet, healthy/
13	“DASH diet *”.mp.
14	“Dietary Approaches to Stop Hypertension*”.mp.
15	“Low-GI Diet *”.mp.
16	“Healthy Diet *”.mp.
17	“Prudent Diet *”.mp
18	“Healthy Eating Ind * mp
19	“Alternative Healthy Eating Ind *”.mp.
20	“Inflammatory diet *”.mp.
21	“Diet* Inflammatory Ind *”.mp.
22	“Food pattern *”.mp
23	Cardiovascular disease
24	Stroke/or Embolic Stroke/or Ischemic Stroke/or Hemorrhagic Stroke/
25	Myocardial Infarction/
26	Coronary Disease/or Cardiovascular Diseases/or ischaemic heart disease.mp. or Myocardial Ischemia/
27	Peripheral Vascular Diseases/
28	Atherosclerosis
29	Glucose intolerance
30	Prediabetic State/ or impaired fasting glucose.mp.
31	Impaired glycaemia.mp
32	impaired glucose tolerance.mp. or Glucose Intolerance/
33	Diabetes Mellitus, Type 2/or Diabetes Mellitus/
34	Hypertension
35	Obesity associated inflammation/or morbid obesity/or diabetic obesity/or obesity
36	Body weight
37	Overweight
38	Dysglycaemia
39	Kidney injury/ or chronic kidney failure/or kidney failure/or acute kidney failure/or kidney disease/or kidney function
40	Renal impairment
41	2 or 3 or 4 or 5 or 6 or 7 or 8 or 9 or 10 or 11 or 12 or 13 or 13 or 14 or 15 or 16 or 17 or 18 or 19 or 20 or 21 or 22
42	23 or 24 or 25 or 26 or 27 or 28 or 29 or 30 or 31 or 32 or 33 or 34 or 35 or 36 or 37 or 38 or 39 or 40
43	1 and 41 and 42
44	Limit 43 to (humans)

### Appendix A.2. Web of Science Search Terms

1	cardiovascular disease (All Fields) or stroke (All Fields) or isch$emic heart disease (All Fields) or coronary disease (All Fields) or myocardial isch$emia (All Fields) or (All Fields) or (All Fields) or atherosclerosis (All Fields) or glucose intolerance (All Fields) or prediabetes (All Fields) or impaired fasting glucose (All Fields) or impaired glucose (All Fields) or impaired glucose tolerance (All Fields) or diabetes mellitus (All Fields) or type 2 diabetes (All Fields) or hypertension (All Fields) or obesity (All Fields) or kidney failure (All Fields) or renal impairment (All Fields) or body weight (All Fields) or overweight (All Fields)
2	ALL = (diet *)
3	gestational diabetes (All fields) or “diabetes in pregnancy” (All Fields)
4	#1 AND #2 AND #3

## Appendix B. Sample Data Extraction Form

(1) Study details and characteristics	
Citation details	
Methods	
Country	
Study design	
Study date	
Study duration	
Number of study centres	
Setting	
Study aim	

Participants	
Study cohort	
Who participated	
Inclusion criteria	
Exclusion criteria	
Subgroup analyses	
Interventions	
Intervention	
How is adherence to dietary pattern recorded?	
GDM criteria used	
Control	
N numbers	
Outcomes	
Primary outcomes	
Secondary outcomes	
Additional outcomes	
Study details	
Length of follow-up	
Trial ID	
Publication details	
Language of publication	
Funding	
Main relevant findings	
