# Supplementary material for: The Association of Specific Dietary Patterns with Cardiometabolic Outcomes in Women with a History of Gestational Diabetes Mellitus: A Scoping Review
Source: Nutrients. 2023 Mar 27;15(7):1613. doi: 10.3390/nu15071613 (PMC10097232; doi:10.3390/nu15071613)
Supplement: Supplementary file 1 [file nutrients-15-01613-s001.zip › nutrients-2284174-supplementary.pdf]

Author, Year of Publication	Evidence Source	Study Design	Country	Dietary Pattern	N number Included in Final Analysis *	Aim	Outcomes of Interest	Key Relevant Findings
Bao, 2015	Original article	Prospective cohort study	USA	Animal/Plant	4502	To examine the long-term risk of T2DM in association with three low-carbohydrate dietary patterns among women with a history of GDM.	T2DM	Overall and animal LCD scores were positively and significantly associated with risk of T2DM among women with a history of GDM, whereas the vegetable LCD score was not significantly associated with increased risk.
Chen, 2022 [a]	Original article	Cluster randomised controlled trial	China	CHEI	160/160	To evaluate the process measures (attendance, engagement, fidelity, and satisfaction) of a tailored intensive lifestyle modification program delivered by local healthcare providers for women with prior GDM in rural China, and to examine the program's 6-month efficacy in reducing diabetes risks compared with the usual care.	FPG, 2-h OGTT, WC, BMI, T2DM risk score, dietary intake	Significant reductions in FPG, 2-h OGTT, WC, and T2DM risk scores of participants in the intervention group compared to the control group. No significant intervention effect on BMI.
Ferranti, 2013	Abstract of conference proceedings	Cross-sectional descriptive study	USA	AHEI	75	To examine the relationship between diet quality as measured by the AHEI and cardiometabolic risk status in women early after a GDM pregnancy.	BMI, HbA1c, metabolic syndrome	Better AHEI diet quality was protective for BMI status, HbA1c, and metabolic syndrome risk.
Franzago, 2018	Original article	Cohort study	Italy	MD	28	To assess the joint predictive role of lipid profiles during	cIMT	A non-statistically significant inverse correlation between the

						pregnancy and of some genetic variants on cIMT taken as a parameter of subclinical atherosclerosis and indicating an early susceptibility to CVD in a cohort of women with a history of GDM.		MD scores with the cIMT values was found.
Ghani, 2014	Original article	Randomised controlled trial	Malaysia	Low GI	39/38	To analyse the effect of dietary GI on FPG, serum lipids, body weight, and body fat of post-GDM subjects with varying fasting serum insulin levels during weight loss.	Changes in weight, total body fat, trunk fat, FPG, TG, HDL, LDL, total cholesterol:HDL ratio, LDL:HDL ratio	Low GI education in women with higher fasting insulin levels resulted in statistically significant improvements in FPG and TG compared to the control group. However, in women with normal/low fasting insulin levels, there were no statistically significant improvements in metabolic parameters.
Gingras, 2012	Original article	Retrospective cohort study	Canada	AHEI	181	To test the hypothesis that women with prior GDM who adopted at least one of the listed preventive practices (regular physical activity, healthy diet, and breastfeeding) were less likely to have altered anthropometric and metabolic profiles compared with women who adopted none of the preventive practices.	BMI, OGTT, Matsuda index, fasting insulin	Higher adherence to AHEI was associated with smaller WC, body fat, fasting insulin, 2 h post OGTT insulinaemia, and Matsuda index. There was a non-significant reduction in BMI and 2-h OGTT and no change in FPG.

Gray, 2021	Original article	Exploratory secondary analysis of a randomised controlled trial	Australia	CSIRO Healthy Eating Score	61/60	To examine whether the weight loss of the participants in a 12-month clinical trial investigating IER compared to CER in women with previous GDM was associated with barriers to weight loss and perception of the risk of T2DM at baseline.<br>To present dietary quality data from the clinical trial and investigate how barriers to weight loss and the perception of diet risk may have influenced the overall diet quality in 12-month IER and CER weight loss intervention in women with a history of GDM.	Dietary quality	CER group showed a weak statistically significant improvement in dietary quality compared with the IER group. CSIRO is not correlated with any metabolic/weight outcomes in this study, but these are reported in the original study (see below).
Gray, 2021 [b]	Original article	Randomised controlled trial	Australia	N/A	32/30	To investigate the effects of a 2-day 500 kcal (2100 kJ) IER regime to CER over 12 months on weight loss and diabetes risk markers in overweight women with previous GDM.	Weight loss, BMI, HbA1c, FPG, fasting insulin, HOMA-IR, 2-h OGTT	Mean weight loss was significant over time but not by diet group. There were no significant between-group differences in change in HbA1c, FPG, fasting serum insulin, HOMA-IR, or 2-h OGTT at 12 months between groups.
Kim, 2011	Original article	Cohort study	Korea	Plant/Animal	381	To determine the early incidence of prediabetes and diabetes after GDM and to identify predictors of postpartum T2DM,	T2DM, prediabetes	Participants who had higher energy, protein, and fat intake from animal sources had higher rates of glucose intolerance and T2DM.

						including anthropometric, biochemical, and nutrient assessments.		
Li, 2015	Original article	Cross-sectional study of ongoing cohort study	USA	AHEI DASH aMED	3818	To examine the associations between long-term adherence to 3 healthy diets with subsequent risk of hypertension among women with a history of GDM, specifically the DASH diet, the alternate Mediterranean diet (aMED), and the AHEI. To determine whether the associations were potentially mediated through changes in BMI.	Hypertension	The cumulative incidence of hypertension among women with AHEI, DASH, and aMED scores in the highest quartile was higher than those in the lowest quartile. Moreover, all 3 dietary pattern scores were strongly inversely associated with hypertension risk. For the AHEI score, 43.9% was mediated through BMI change.
Li, 2019	Original article	Cohort study	USA	AHEI	2434	To examine the association of a genetic risk score for T2DM with the risk of T2DM among two independent populations of women with a history of GDM followed over a long period. To further investigate how this association might be modified by non-genetic factors of T2DM.	T2DM	For the Nurses' Health Study II cohort, the post-partum AHEI score was significantly lower in those who developed T2DM compared to those who did not. For the Danish National Birth Cohort, there was no significant difference in AHEI score between those who developed T2DM compared to those who did not.
Li, 2020	Original article	Baseline survey of ongoing RCT	China	CHEI	404	To evaluate the diet quality of women with previous GDM in two counties of Hunan Province by applying CHEI and to identify influential factors.	Dietary quality and potential influential factors	Overall CHEI scores were low. The distribution of CHEI levels was similar across BMIs.

Li, 2021 [a]	Original article	Randomised controlled trial	China	CHEI MDD-W	138/149	To analyse the effect of a lifestyle intervention on diet quality.	CHEI score, proportion of participants reaching MDD-W, energy and nutrient intake	The intervention group achieved a higher CHEI score and higher MDD-W proportion at 18 months. The lifestyle intervention was associated with the change of CHEI but not with MDD-W.
Louie, 2013	Original article	Follow-up study following interventional RCT	Australia	Low GI	33/25	To investigate the effects of a low-GI diet during pregnancy complicated by GDM on early post-natal outcomes (anthropometric assessment, glucose tolerance test, and lipid profile), in comparison to a conventional high-fibre diet.	Pregnancy outcomes, neonatal anthropometry, maternal metabolic profile:FPG, fasting insulin, 1-h OGTT, 2-h OGTT, HOMA-IR, lipid profiles	No statistically significant differences in relevant outcome measures were found between groups. There was a non-significant trend toward improved insulin sensitivity, lower TG, and higher HDL in the low GI group.
Mercier, 2019	Original article	Cross-sectional analysis within a cohort study	Canada	FV intake	281	To investigate the association between fruit and vegetable (FV) intake and abnormal glucose tolerance among women with prior GDM.	OGTT, HbA1c, HOMA-IR, Matsuda index, insulinogenic index	Women with abnormal glucose tolerances had significantly lower FV and vegetables servings and tended to have lower fruit servings than women with NGT. FV intake was associated with a reduced likelihood of having abnormal glucose tolerance. In women with abnormal glucose tolerances, vegetable intake was negatively correlated with HOMA-IR and positively correlated with Matsuda index

								after adjustment for age and BMI. There was a non-significant tendency toward similar associations with combined fruit and vegetable intake, and there was no association with fruit intake.
Morrison, 2012	Original article	Cross-sectional study	Australia	ARFS	1447	To describe the diet quality of a national sample of Australian women with a recent history of GDM and determine factors associated with adherence to national dietary recommendations.	Diet quality, lipid profile, blood pressure, T2DM, BMI	There was no significant difference in rates of hyperlipidaemia, hypertension, or T2DM between each of the quintiles of AFRS adherence. There was a trend such that women with a higher BMI were less likely to be in the highest compared to the lowest ARFS quintile, but this did not reach statistical significance.
Nicklas, 2020	Original article	Follow-up cohort study following "balance after Baby" RCT	USA	Low GI	75	To analyse and deduce the predictors of weight loss in post-partum GDM pregnancies.	Body weight, breastfeeding status, Edinburgh Postnatal Depression Scale, sleep, Harvard Food Frequency, International Physical Activity Questionnaire	Glycaemic index was not associated with levels of post-partum weight loss.

Ott, 2020	Original article	Cross-sectional study	Germany	Low GI	48	To investigate whether mothers with GDM and their offspring show similar metabolomic patterns several years postpartum.	BMI, Matsuda insulin sensitivity index, dietary intake, physical activity	No significant correlations were observed between dietary glycaemic load or physical activity and BMI.
Perez-Ferre, 2015	Original article	Interventional RCT	Spain	MD	126/111	To investigate whether a Mediterranean pattern lifestyle intervention and a monitored program of physical activity can reduce the development of glucose disorders three years postpartum in women who have previously presented with GDM under the real conditions of clinical practice.	Glucose disorders, BMI, WC, fasting insulin, HOMA-IR, lipids, blood pressure HbA1c, physical activity score, nutrition score, fat scores	Non-statistically significant trends towards a reduction in T2DM/IFG/IGT in the intervention group. Significant reductions in BMI, waist circumference, fasting plasma insulin, HOMA-IR, LDL-cholesterol, TG, and Apo lipoprotein B observed in the intervention group compared with the control group. These changes were not correlated directly with adherence to the Mediterranean nutritional pattern; however, there was significantly higher adherence to this nutritional pattern in the intervention group compared to the control group at 3 years.
Shin, 2017	Abstract	Cohort study	USA	DII	176	To explore the effect modification of history of GDM on the association between the Dietary Inflammatory Index and beta-cell function and insulin resistance using homeostatic	HOMA-IR	Among the women without a history of GDM, women who were in the highest (most pro-inflammatory) tertile of DII scores had increased risk for insulin resistance compared to the lowest (most anti-inflammatory) tertile of the DII

						model assessment for insulin resistance (HOMA-IR).		(adjusted odds ratios = 1.56; 95% CI = 1.07–2.27). However, no such association was observed among the women with a previous GDM diagnosis.
Shyam, 2013	Original article	Interventional RCT	Malaysia	Low GI	77	To evaluate the effects of conventional dietary recommendations administered with and without additional low-GI education, in the management of glucose tolerance and body weight in Asian women with previous GDM.	FPG, OGTT, fasting insulin, anthropometric measures	Low GI yielded statistically significant reductions in bodyweight, BMI, waist:hip ratio, and 2-h OGTT.
Tang, 2021	Original article	Prospective cohort study	China	Plant/Animal	104 with GDM	To examine the association between LCD during puerperium and FPG and risk of IFG in the early postpartum period, and its interaction with history of GDM, as well as how LCD scores are associated with the whole distribution of FPG using quantile regression models.	FPG	There was a significantly positive association between animal LCD score and FPG in women without GDM, but not in their counterparts with GDM.
Tobias, 2012	Original article	Prospective cohort study	USA	AHEI<br>DASH<br>aMED	4413	To quantify the association of adherence to these healthful dietary patterns and T2DM risk among women with a history of GDM by means of	T2DM	All 3 dietary pattern adherence scores were strongly and inversely associated with T2DM risk after adjusting for age and total energy intake.

						prospective follow-up of 16 years.		
Tobias, 2016	Original article	Prospective cohort study	USA	AHEI DASH aMED	3397	To assess the relationship between change in adherence to three healthful dietary patterns: the AHEI, DASH, and aMED, and long-term change in weight, to determine whether improved diet quality may provide one strategy for the prevention of weight gain among women with a history of GDM, prior to the development of T2DM.	Body weight	Women in the highest quintile of diet change (most improvement in quality) gained significantly less weight per 4-year-period than the lowest quintile, independent of other risk factors.

Supplementary Table S1. Overview of the characteristics, aims, and main findings of included studies.

AHEI: Alternate Healthy Eating Index, MD: Mediterranean diet, aMED: alternate Mediterranean diet, DASH: Dietary Approaches to Stop Hypertension, Chinese Healthy Eating Index, CSIRO: Commonwealth Scientific and Industrial Research Organisation, ARFS: Australian Recommended Food Score, MDD-W: Minimum Dietary Diversity for Women, LCD: Low-carbohydrate diet, GI: Glycaemic Index, DII: Dietary Inflammatory Index, IER: intermittent energy restriction, CER: continuous energy restriction, FV: fruit and vegetable, GDM: Gestational Diabetes Mellitus, T2DM: Type 2 diabetes mellitus, IFG: impaired fasting glucose, IGT: impaired glucose tolerance, FPG: fasting plasma glucose, 2-h OGTT: glucose 2 h after oral glucose tolerance test, WC: waist circumference, BMI: body mass index, HbA1c: glycated haemoglobin, cIMT: carotid intima media thickness, TG: triglyceride, HDL: high-density lipoprotein, LDL: low-density lipoprotein, HOMA-IR: homeostasis model assessment of insulin resistance. * Single figure for total in cohort studies, two figures refer to intervention N/control N for interventional studies. [a] These articles are reports of the same cohort. Whilst no one article included information regarding dietary pattern and metabolic outcomes, information from all articles was used to draw inferences about metabolic outcomes and low GI adherence in the cohort. [b] This article was not included in the original search but was a report of the original randomised controlled trial referenced in [69]. Information regarding metabolic outcomes from this article was used to evaluate the relationship between outcomes and dietary pattern as reported in the included article.
